# Merit of integrating in situ transcriptomics and anatomical information for cell annotation and lineage construction in single-cell analyses of *Populus*

**DOI:** 10.1186/s13059-024-03227-5

**Published:** 2024-04-03

**Authors:** Ying-Lan Chen, Jo-Wei Allison Hsieh, Shang-Che Kuo, Chung-Ting Kao, Chia-Chun Tung, Jhong-He Yu, Tien-Hsien Chang, Chuan Ku, Jianbo Xie, Deqiang Zhang, Quanzi Li, Ying-Chung Jimmy Lin

**Affiliations:** 1 Department of Biotechnology and Bioindustry Sciences, College of Bioscience and Biotechnology, National Cheng Kung University, Tainan, Taiwan; 2 Department of Life Science, National Taiwan University, Taipei, Taiwan; 3 Genome and Systems Biology Degree Program, National Taiwan University and Academia Sinica, Taipei, Taiwan; 4 Institute of Plant Biology, National Taiwan University, Taipei, Taiwan; 5 Genomics Research Center, Academia Sinica, Taipei, Taiwan; 6 Institute of Plant and Microbial Biology, Academia Sinica, Taipei, Taiwan; 7 National Engineering Laboratory for Tree Breeding, College of Biological Sciences and Technology, Beijing Forestry University, Beijing, China; 8 Key Laboratory of Genetics and Breeding in Forest Trees and Ornamental Plants, Ministry of Education, College of Biological Sciences and Technology, Beijing Forestry University, Beijing, China; 9 State Key Laboratory of Tree Genetics and Breeding, Chinese Academy of Forestry, Beijing, China

## Abstract

Cell type annotation and lineage construction are two of the most critical tasks conducted in the analyses of single-cell RNA sequencing (scRNA-seq). Four recent scRNA-seq studies of differentiating xylem propose four models on differentiating xylem development in *Populus*. The differences are mostly caused by the use of different strategies for cell type annotation and subsequent lineage interpretation. Here, we emphasize the necessity of using in situ transcriptomes and anatomical information to construct the most plausible xylem development model.

**Supplementary Information:**

The online version contains supplementary material available at 10.1186/s13059-024-03227-5.

## Main text

The application of single-cell RNA sequencing (scRNA-seq) opens a new era for scientists to explore dynamic cell trajectories. One of the biggest challenges of scRNA-seq analyses is the cell type annotation. The first step of scRNA-seq involves cell isolation from the tissue/organ of interest to obtain their transcriptomic profiles. Such isolation usually leads to the loss of morphological information, which complicates subsequent cell type annotation. The use of marker genes is the most common strategy for cell type annotation in scRNA-seq analyses [1]. In plants, numerous marker genes identified by high-throughput spatiotemporal manners in *Arabidopsis* were used for the cell type annotation [2, 3]. This high-throughput profiling remains largely unexplored in almost all other non-*Arabidopsis* plant species, which restricts the availability of reliable marker genes.

A common strategy for cell type annotation in non-*Arabidopsis* species is the use of the orthologs of the marker genes from *Arabidopsis*, which heavily relies on the functional conservation between *Arabidopsis* and other species. However, many studies have reported the discrepancy on tissue development and orthologous gene expression patterns between *Arabidopsis* and other species [4, 5]. Take xylem development for example, *Arabidopsis* lacks one xylem cell type (ray parenchyma) and the development of another xylem cell type (libriform fiber) is incomplete with respect to woody eudicots [6]. The expression patterns of many genes involved in xylem development are also different between *Arabidopsis* and other woody species, such as *Populus* [4–6]. This functional and developmental diversity underscores the risks of using *Arabidopsis* marker genes for cell type annotation in woody eudicots.

**Fig. 1:**
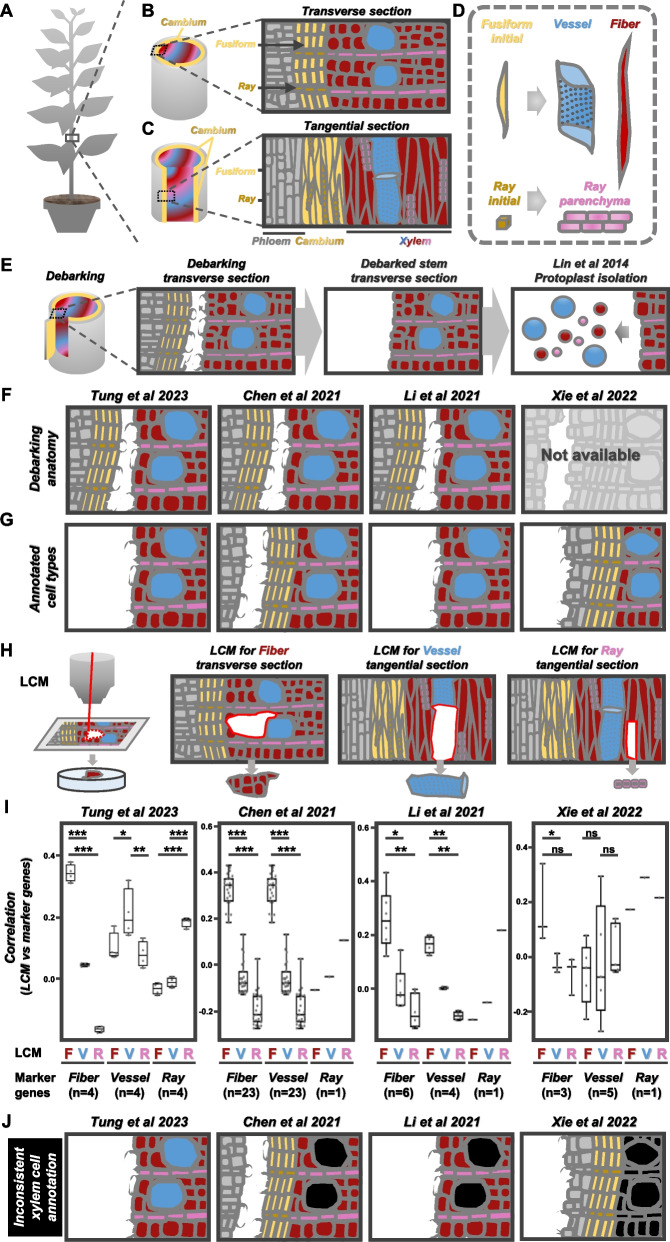
Xylem anatomy and cell type annotation. **A**–**C** Schematics of a woody plant (**A**), the cambium (fusiform and ray initials) on the transverse section (**B**), and the tangential section (**C**) of the stem. **D** Xylem cell morphologies via axial and radial systems. Vessel elements (labeled as vessel) and libriform fibers (labeled as fiber) both derived from fusiform initials. Ray parenchyma cells derived from ray initials. **E** Debarking and differentiating xylem protoplast isolation. **F** Anatomical analyses during stem debarking of four previous studies (shown as Tung et al. 2023 [6], Chen et al. 2021 [10], Li et al. 2021 [9], and Xie et al. 2022 [8]). The anatomical data is not available in Xie et al. **G** Annotated cell types on stem after debarking of four previous studies. **H** Schematics of laser capture microdissection (LCM) procedure to harvest three cell types for in situ cell transcriptomes, including libriform fiber (red area), vessel element (blue area), and ray parenchyma cell (pink area). **I** The correlation was obtained by the following two steps: (i) first round correlation analyses between the in situ cell transcriptomes and scRNA-seq transcriptomes. For each cell in scRNA-seq, a correlation coefficient was obtained. (ii) Second round correlation analyses between the results from (i) and the transcript abundance of each marker gene in each cell. The correlation (shown as LCM vs marker genes) were illustrated as the results from second round correlation analyses. F, libriform fibers. V, vessel elements. R, ray parenchyma cells. One, two, and three asterisks represent Student’s *t*-test *p* < 0.05, 0.01, and 0.001, respectively. ns, no significant difference. **J** Inconsistent xylem cell type annotations (shown as black area) (Chen et al. for vessel elements; Li et al. for vessel elements; Xie et al. for libriform fibers, vessel elements, and ray parenchyma cells)

During xylem development, stem cells (initials) proliferate into proximal cambium to produce differentiating xylem with two architectural systems, axial system (growing upward and downward) and radial system (growing inward and outward) [7]. Each system possesses its own stem cells as fusiform initials and ray initials (Fig. 1A–C). These initial cells and their proliferating descendant cells are called vascular cambium (Fig. 1B–C). Differentiating xylem is composed of three cell types as vessel elements, libriform fibers (both developed from fusiform initials), and ray parenchyma cells (from ray initials) (Fig. 1D). Four recent studies reported the developmental lineages of differentiating xylem in poplar through scRNA-seq analyses [6, 8–10] using the differentiating xylem protoplasts isolated by the same pipeline [11]. The first step of such protoplasting pipeline is stem debarking. Anatomical analyses showed an obvious separation of vascular cambium and differentiating xylem after debarking, and the bark contains phloem and vascular cambium (Fig. 1E). The subsequent protoplast isolation then allows the collection of differentiating xylem protoplasts (Fig. 1E) [6, 8–11]. Such anatomical results after debarking are very consistent among different species grown in various regions, such as North America, East Asia, and Australia [6, 9, 11, 12]. Among the four studies, Tung et al. [6], Chen et al. [10], and Li et al. [9] used anatomical analyses to ensure the debarking effect as the separation of cambium and differentiating xylem (Fig. 1F). In Chen et al. [10], the cambium was located on the bark side based on the anatomical results, but vast majority of the cells in the “cambium region” clusters resided on the debarked stem using the cell type annotation results, showing the challenges in cell type annotation on the basis of the scRNA-seq data. The anatomical analyses of debarking effect were not reported in Xie et al. [8] (Fig. 1F). Upon the use of the same protoplasting pipeline, the scRNA-seq results between Xie et al. [8] and Tung et al. [6] showed extremely high overlapping rates (99.9%, Additional file 1), which demonstrates the highly similar debarking effects between these two studies. The cell type annotation of Xie et al. [8] on debarked stem included cambium and phloem and conflicted to the suggested debarking effect (Fig. 1F, G). These results highlight the importance of careful anatomical inspection of the samples, in which the separation of tissues by peeling provides critical information on the cell types that can be present in the scRNA-seq data.

Due to the lack of reliable marker genes in differentiating xylem, Tung et al. [6] generated in situ cell transcriptomes using laser capture microdissection for all three cell types in differentiating xylem (Fig. 1H) to conduct the annotation. Li et al. [9] and Chen et al. [10] used a mixture of marker genes from poplar and *Arabidopsis* for differentiating xylem cell type annotation. As a result, the marker genes used from both studies (Li et al. [9] for vessel elements; Chen et al. [10] for xylem cells composed of at least vessel elements and libriform fibers) unexpectedly annotated libriform fibers (Fig. 1I). Although Xie et al. [8] used all marker genes from poplar, their marker genes for libriform fibers annotated both libriform fibers and ray parenchyma cells, their vessel element markers annotated all three xylem cell types, and their ray parenchyma marker annotated vessel elements (Fig. 1I). The problems in cell type annotation of these three studies [8–10] (Fig. 1J) demonstrate the needs of in situ cell transcriptomes for the cell type annotation.

**Fig. 2:**
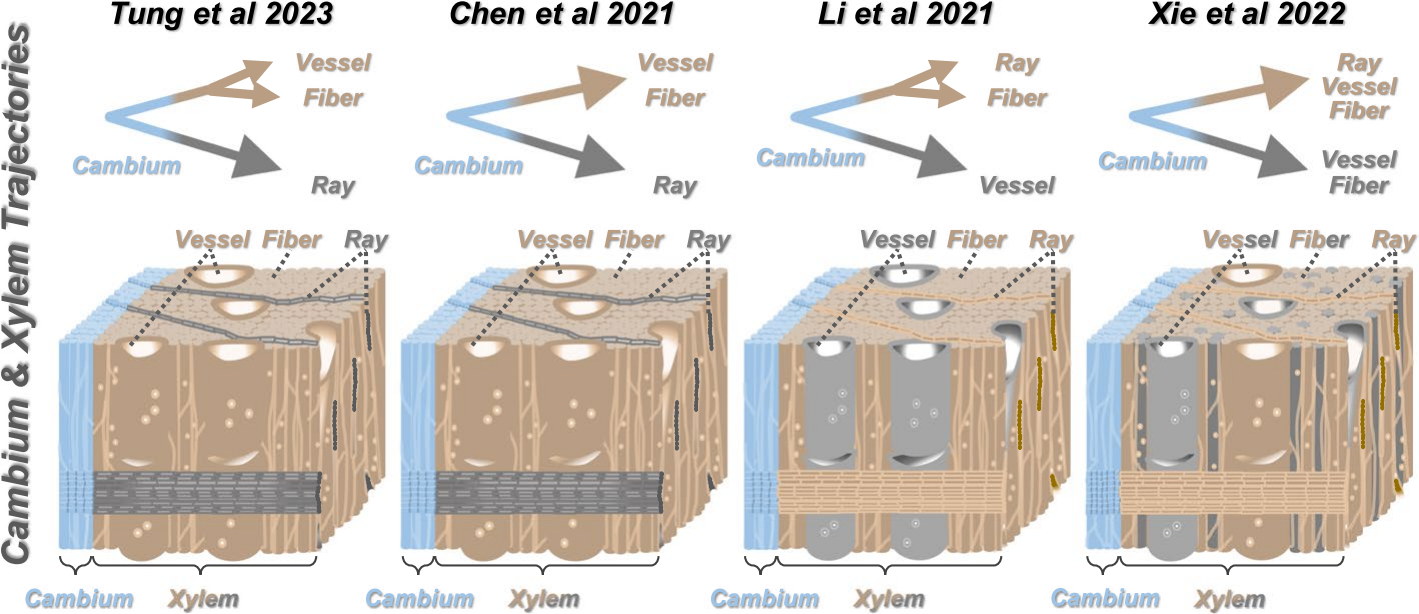
Proposed cell lineages for xylem development. Schematics of proposed developmental cell lineages from vascular cambium to differentiating xylem of the four previous studies (shown as Tung et al. 2023 [6], Chen et al. 2021 [10], Li et al. 2021 [9], and Xie et al. 2022 [8]). Blue color represents vascular cambium. Brown and gray colors represent two distinct developmental lineages

Because of the cell type annotation differences, four different models were proposed on the differentiating xylem developmental lineages [6, 8–10]. Starting from vascular cambium, Tung et al. [6] separated fusiform and ray lineages and further divided fusiform lineages into vessel elements and libriform fibers (Fig. 2), whereas Chen et al. [10] merged the lineages of vessel elements and libriform fibers together (Fig. 2). In the other two studies, Li et al. [9] and Xie et al. [8] both mixed ray parenchyma lineage with libriform fibers or even vessel elements (Fig. 2). Previous anatomical analysis [13, 14] unambiguously showed that axial and radial systems are architecturally independent, thus rendering the proposed models by Li et al. [9] and Xie et al. [8] almost impossible. Taken together, the model proposed by Tung et al. [6] would be the most plausible one, on the basis of the available scientific data, for explaining differentiating xylem development.

This analysis highlights the necessity of using in situ cell transcriptomes for cell type annotation. Few marker genes from either the source species or the homologs of *Arabidopsis* would be inadequate for cell type annotation. The inconsistency of the models among the four studies on developmental lineages demonstrates the importance of the incorporation of anatomical knowledge. Mathematical algorithm-based reconstruction using scRNA-seq data may not faithfully describe the real-world biological processes. The combination of anatomical data and in situ transcriptomes coupled with scRNA-seq analyses would lead to a more reliable conclusion.

### Supplementary Information

**Additional file 1.** Supplementary figure S1.

**Additional file 2.** Review history.

## Data Availability

Sequence data was downloaded from the four previous studies [6, 8-10]. For scRNA-seq data analysis, the raw reads from Tung et al. [6] were downloaded from National Center for Biotechnology Information (NCBI) under accession number GSM5453537. The raw reads from Xie et al. [8] were downloaded from National Genomics Data Center under BioProject accession number PRJCA014789. The raw reads from Li et al. [9] were downloaded from NCBI under BioProject accession number PRJNA703312. The raw reads of wood tissues in Chen et al. [10] were downloaded from National Genomics Data Center under BioProject accession number PRJCA005543. For lcmRNA-seq data analysis, the raw reads from three replicates of libriform fibers, vessel elements, and ray parenchyma were downloaded from NCBI under accession number GSE180121.
