# Supplementary material for: Merit of integrating in situ transcriptomics and anatomical information for cell annotation and lineage construction in single-cell analyses of Populus
Source: Genome Biol. 2024 Apr 3;25:85. doi: 10.1186/s13059-024-03227-5 (PMC10988922; doi:10.1186/s13059-024-03227-5)
Supplement: Supplementary file 1 — Additional file 1. Supplementary figure S1. [file 13059_2024_3227_MOESM1_ESM.docx]

**Overlapping rates between cells from Tung et al and the other three studies**

The quantification of the overlapping rate of distribution between the cells from Tung et al (indicated as “Tung et al 2023” in the figure below) and each one of the other three studies (indicated as “Chen et al 2021”, “Li et al 2021”, “Xie et al 2022” below) was performed using minimal spanning trees (MSTs), as developed in Tung et al. The MSTs were constructed based on integrated UMAP plots, and the subgraphs were derived by eliminating edges connecting cells from different studies. The central node in each subgraph was determined by identifying the node with the highest closeness centrality. The processes of MST construction, graph determination, and closeness centrality computation were executed using igraph in R. The density of these center nodes was calculated as follows. The distribution of these center nodes was separated into individual studies for the calculation of densities by MASS. The densities from each study were weighted by the number of center nodes and the proportion of cells, and these weighted densities were then combined to form a concatenated density. A concatenated density was normalized by the total density of the cells from Tung et al. Densities ranging from 0 to 1 were segmented into 500 bins with different color shading. The proportions of different densities were visualized in a pie chart. The overlapping rate of distribution was determined by sum of the proportions, excluding the lowest bin (i.e., cells not overlapping between the two studies), and is indicated next to the pie chart. As a result, the overlapping rates of the scRNA-seq results using the protoplasts isolated from debarked stem were extremely high (Tung and Chen “wood” = 96.7%; Tung and Li = 99.5%; Tung and Xie = 99.9%).


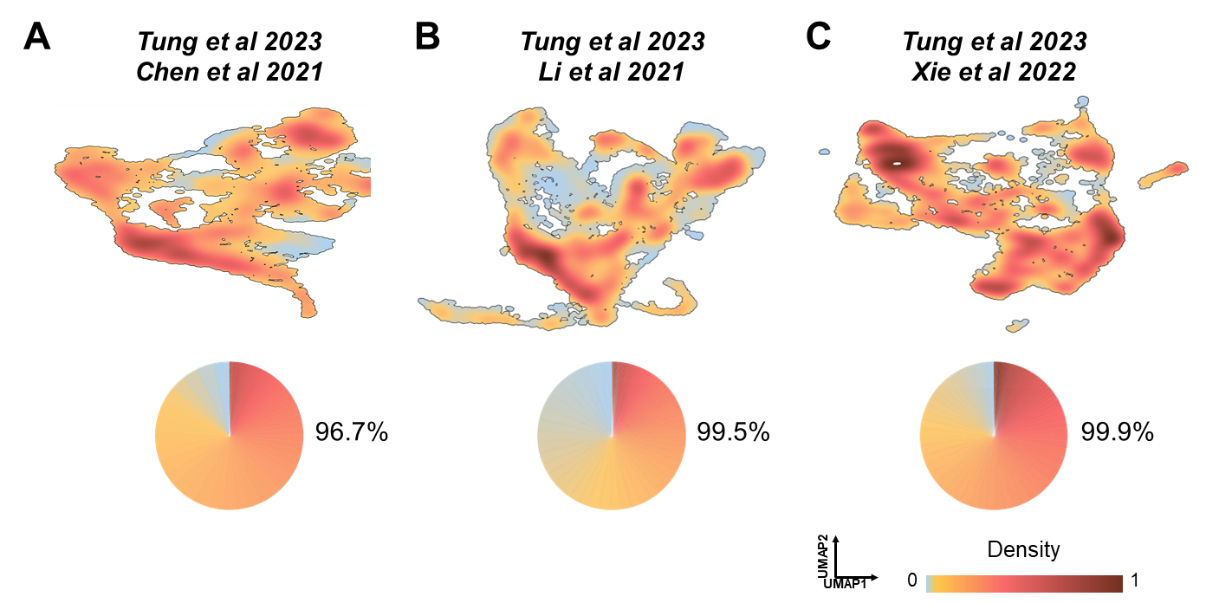


**Supplementary figure S1.** The overlapping rates of the scRNA-seq results using the protoplasts isolated from debarked stem. (A) Tung et al 2023 and Chen et al 2021. (B) Tung et al 2023 and Li et al 2021. (C) Tung et al 2023 and Xie et al 2022.
