# Supplementary material for: Merit of integrating in situ transcriptomics and anatomical information for cell annotation and lineage construction in single-cell analyses of Populus
Source: Genome Biol. 2024 Apr 3;25:85. doi: 10.1186/s13059-024-03227-5 (PMC10988922; doi:10.1186/s13059-024-03227-5)
Supplement: Supplementary file 2 — Additional file 2. Review history. [file 13059_2024_3227_MOESM2_ESM.docx]

Review History

**First round of review**

**Reviewer 1**

**Were you able to assess all statistics in the manuscript, including the appropriateness of statistical tests used?**

There are no statistics in the manuscript.

**Comments to author:**

The commentary deals with a very important aspect of single cell RNAsequencing in Populus stem. It focuses on methods to annotate and validate the identity of differentiating xylem elements from single cell RNA sequencing data. Data from four different published papers are compared and commented. I have a few concerns on the methodology and the conclusions presented in this commentary.

The manuscript concludes that the xylem cell identities and developmental trajectories presented in Tung et al is the only correct one. I think it would have been more fruitful to discuss and compare the different methods used in the four studies, such as in situ hybridisation, reporter gene lines, spatial transcriptomics and laser dissection, to validate the marker genes (and the identity of the clusters). What are the advantages and disadvantages of each method? Do the authors perhaps have a recommendation on the methodology? Is it possible to propose a set of good marker genes for the different xylem cell types on the basis of these works? This would be most helpful for the community.

The conclusion on Tung et al having the most correct cell annotations and hence xylem trajectories is based on a new analysis of data published in the four papers. In this analysis they correlate the expression of marker genes used in three of the articles to their expression reported in three samples, collected by laser capture microdissection (LCM), in Tung et al. This analysis is based on the assumption of the authors that the laser dissection method is the ultimate method/proof and the only reliable method to validate cell identities. However, LCM has inherent problems. First of all, there is a risk of including unwanted neighbouring cells (or cells below) from the sections during LCM. Trials by others have failed to provide RNA from the intended cell types due to production of heat by the laser (e.g. Larisch et al 2012 Plant Physiology 160: 1515-1529), which is many times overcome by increasing the size of captured area (and losing the cell specificity). It is also difficult with the LCM technique to cover exactly the same area as obtained with the SDX protoplasting. Tung et al includes cells for LCM from 20 cell layers of cryosections, while the schematic figure 1A in Tung et al indicates that SDX protoplasts were derived from eight cell layers. Furthermore, it is difficult if not impossible, to define in the cryosections whether the fast-differentiating cells, such as vessel elements, are viable. In Tung et al, it seems from the data analysis in figure 1J that the LCM vessel sample represents transcription of genes that can be found in several scRNAseq cell clusters. Does it mean that the LCM sample represents several cell types or perhaps both fibers and vessels at different developmental stages, or does it mean that vessel elements (at different stages of development) are present in several clusters in the scRNAseq data? Fig. S7 in Tung et al also shows that there is no correlation between gene expression in the LCM vessel sample with any of the scRNAseq clusters, rendering the true identity of the vessel LCM and/or the annotation of the clusters unclear. Looking more carefully in the Reviewer files of Tung et al (Additional file 17), it seems that the LCM sampling was done very carefully, and that the gene expression results from their LCM data on vessels and fibers correspond very well to previously published RNAseq data on laser microdissected fibers and vessels. This makes me confident with the LCM data of Tung et al but at the same time questions the correct interpretation of their scRNAseq clustering. It says in the Additional file 17 in Tung et al for instance that "In this study, XCP2 is enriched in libriform fibers in the scRNAseq results, but is enriched in vessel elements in the lcmRNAseq results". It would be really interesting for the community working with this technology to know what could be the reason(s) behind this, especially as XCP2 is very commonly used as a marker gene in scRNAseq analyses. My guess is that the cell and tissue composition in the laser microdissected samples and in the protoplast population is different. For instance, it is likely that differentiating vessel elements are poorly represented in the scRNAseq data due to their large size that is incompatibility with both the protoplasting method and the chromium technology, which is why vessels expressing XCP2 are not enriched in cluster 1 in Tung et al. Why XCP2 expressing fibers are not enriched in the LCM fiber sample, is more difficult to explain. Any comments on all these topics?

The commentary makes several unmotivated claims on misinterpretation of the data in Chen et al. First, it is claimed on lines 87-89 that Chen et al misinterpreted the effect of debarking. This is however not the case. They show in supplementary figure S1A very clear microscopy images on the breaking point after peeling of the bark, which does not correspond to what is drawn in Figure 1F and G in the current manuscript. In Chen et al, the breaking point is not important either, and does not influence the conclusions since scRNAseq data from the bark and the secondary xylem were pooled for the data analysis. Please correct (and redraw in figure 1) this part concerning Chen et al.

Second, it is claimed on lines 96-97 on the basis of the correlation analysis in figure 1I that Chen et al annotated "vessel elements/libriform fibers unexpectedly as libriform fibers". I cannot however find anywhere in Chen et al. information on any marker genes specifically for fibers and vessels. They reported throughout the manuscript results on only "xylem elements" since obviously they did not find evidence in separating the developmental trajectories for fibers and vessels. So it is not surprising that their xylem marker genes (that were not claimed to be specific to fibers or vessels) correlated with the localisation of the LCM sample of fibers. Apparently the markers they used were mostly expressed in the fibers. This needs to clarified and correctly reported in the commentary, and corrected in figure 1I.

Third, it is claimed in lines 107-109 that annotation of vascular cambium and ray parenchyma cells were misplaced in Chen et al. The evidence for this claim does not become clear from Tung et al, and should be clarified here thoroughly.

Minor comments

A correlation analysis between marker genes in Tung et al (for instance marker genes shown in Sfigure21 and 22) and the LCM data needs to be added to figure 1I, similar to what is shown for the other three papers.

Since one the aims with the commentary is to put focus on correct annotation of the different xylem cell types and correct anatomical knowledge, it is important the authors adhere to well established nomenclature on the cell types and their differentiation in the cambial zone (according to the references Larson 2012 and Evert 2006).

Lines 69-72. The term "fusiform system" is artificial, and definitely not widely, if at all, used. Vascular cambium contains fusiform initials that are named "fusiform" due to the origin of the word (shape that is broad in the middle and narrows down towards the tip). I strongly discourage inferring terms to the literature that are confusing and that don't correspond to the etymology of the word "fusiform". Furthermore, vascular cambium does not differentiate or grow in any direction , but produces xylem precursor cells inwards in the stem. Cells don't either differentiate into stem differentiating xylem (SDX) but into xylem fibers, vessels or parenchyma. Please reformulate this part.

The use of the term "SDX" is not justifiable in this context. Only two of the papers uses this term. Please use instead the more simple term "differentiating xylem".

it is discussed in lines 79-85 that the debarking separates vascular cambium and "SDX", and that it is very consistent among different species grown in various regions. This is an oversimplification since cambial cell divisions rates influence the breaking point in the cambial region when debarking. But clearly the most fragile area is the expanding xylem elements, resulting in a rupture in this tissue during debarking. However, it cannot be excluded that cambial cells sometimes rip off along with the secondary xylem tissues. This is supported by frequently observed expression of not only the cambial markers but also genes related to cell division related and even phloem precursor identity. Therefore, talking about SDX as if it was always reproducibly collected and/or well defined is misleading.

The authors are however completely correct in that it is important (and helpful for the data interpretation) to know where the debarking occurs. However, I would like to point out that an image on a 300 um wide cross section (as in Li et al.) or schematic pictures (as in Tung et al.) are not enough to demonstrate the fracture site and do not allow excluding for instance cambial cells ripping off together with the differentiating xylem.

Lines 97-91. Chen did not misinterpret the anatomy of debarked stem. Xie et al. does not give information in the paper on where the bark is detached, and it is difficult to see how their misinterpretation could have led to "subsequent mis-annotation of SDX cells". They define a phloem cluster, and without looking at the details of this cluster, I would not disclose the possibility that this cluster is not composed of phloem precursor cells. This is important for the reader to know. Is it certain that the phloem cluster in Xie et al is not correct, as claimed on lines 90-91? If no better evidence is provided on misintepretation in the cell composition of the sample (influenced by the fracture site), please remove the focus on the sampling method in figure 1.

One more paper has been published since the submission of this commentary. Please include descriptions and analysis of this manuscript as well.

The English language should be checked. For instance lines 46-48, 51-54, and 56.

**Reviewer 2**

**Were you able to assess all statistics in the manuscript, including the appropriateness of statistical tests used?**

There are no statistics in the manuscript.

**Comments to author:**

The power of single-cell technologies in plant biology research has been remarkable, with applications expanding beyond Arabidopsis to other plant species. However, a standardized paradigm for the application of this new technology is yet to be established. In this correspondence, valuable insights are presented for current single-cell studies in plant developmental research. Integrating in situ transcriptomics and anatomical information in single-cell analyses holds great promise for precise cell annotations. Using a previous study as an example, the authors compare key findings on xylem development with three other single-cell studies. Through this comparison, they highlight potential mis-annotations of cell types in previous studies and emphasize the necessity of combining in situ transcriptomics and anatomical information in single-cell analyses. Overall, the manuscript is well-written and the presents a thought-provoking perspective.

**Authors Response**

**Point-by-point responses to the reviewers’ comments:**

**Reviewer #1**

The commentary deals with a very important aspect of single cell RNA sequencing in Populus stem. It focuses on methods to annotate and validate the identity of differentiating xylem elements from single cell RNA sequencing data. Data from four different published papers are compared and commented. I have a few concerns on the methodology and the conclusions presented in this commentary.

*----- [Author Response] -----*

*We thank the Reviewer for valuable comments on our manuscript. For the ease of reading, we labeled the Review Concerns by numbers, such as Major Concerns (1), (2) and (3). The nomenclature of cell clusters in this Document represents the working hypothesis for the developmental paths of differentiating xylem in Tung et al. We have listed our point-by-point responses to all comments and suggestions below.*

Major Concerns:

**(1)** The manuscript concludes that the xylem cell identities and developmental trajectories presented in Tung et al is the only correct one. I think it would have been more fruitful to discuss and compare the different methods used in the four studies, such as in situ hybridisation, reporter gene lines, spatial transcriptomics and laser dissection, to validate the marker genes (and the identity of the clusters).

*(1-1)* What are the advantages and disadvantages of each method?

*----- [Author Response] -----*

***In situ hybridization***

*Advantage: This method can detect endogenous mRNA.*

*Disadvantage: The specificity of the probes is difficult to verify and interpret. Synthetic probes are used to hybridize the endogenous mRNA in plant tissue sections. This method can directly identify the endogenous transcribed mRNA, but suffer from the unknown specificity between probes and endogenous mRNA (PMID: 21276820; PMID: 2023940). The specificity could be theoretically improved using the unique sequences on the transcript of interest for probe design. However, numerous practical issues would occur, such as: (i) The “unique sequence” is defined by the current assembled genome sequence with current annotated transcript sequence. With the improvement of sequencing technology, genome sequence and annotated transcript sequence have been revised and updated frequently. (ii) The abundance of each transcript varies dramatically. For the probes, their hybridization against the endogenous transcripts would exhibit as “more or less” specific instead of “yes or no”. For example, a probe is 100% similar to a region on a transcript, but may be 40% similar to another region on another transcript. Transcripts with low specificity would still be able to be hybridized by the probes due to their high abundance, and transcript with high specificity would not be able to be hybridized due to their low abundance. Thus, the abundance of endogenous transcripts would interfere the hybridizing effects of the probes. The specificity issues are difficult to be excluded during in situ hybridization, leading to the risk on identifying unwanted cells and ignoring targeted cells (please see below for Response Fig. 1A).*

***Reporter gene lines***

*Advantage: Plant sectioning may not be required. Signals could be observed in living plants.*

*Disadvantage: Reporter gene lines strategy uses the promoter from gene of interest to drive green fluorescence protein or β-glucuronidase (GUS) as reporters. The length of the promoters is basically defined arbitrarily, and promoters with different length would result in different* *expression patterns. For example, different promoter length of WUSCHEL resulted in different expression pattern (PMID: 15980263). Thus, the consistency on expression pattern between endogenous genes and report genes remained difficult to be assessed, which leads to the similar issue as in situ hybridization on the “unwanted/targeted” cells (please see below for Response Fig. 1B).*

***In situ transcriptome by laser capture microdissection (LCM)***

*Advantage: This method can detect endogenous mRNA. The “signal” from RNA sequencing is much more reliable than those from in situ hybridization and reporter gene line. The signals from in situ hybridization and reporter gene line are basically interpreted by colorimetric test or fluorescence. For example, the regions generated blue color, purple color or fluorescence would be defined as the expressing locations. RNA sequencing generates signals by providing specific information as nucleotide sequences followed by rigorous statistical analyses to eliminate false signals. In situ transcriptome by LCM (later as lcmRNA-seq) then could be regarded as the most precise platform for cell-type annotation among these three methods (please see below for Response Fig. 1C). Furthermore, the transcript abundance can be quantified.*

*Disadvantage: The procedure of LCM is extremely labor intensive. In addition, it may require years of cell collection for harvesting sufficient cell amount for transcriptomic analyses.*

***Response Figure 1.*** *Comparison of different methods for marker gene validation. (A) In situ hybridization. (B) Reporter gene line. (C) In situ transcriptome.*

*(1-2)* Do the authors perhaps have a recommendation on the methodology?  Is it possible to propose a set of good marker genes for the different xylem cell types on the basis of these works? This would be most helpful for the community.

*----- [Author Response] -----*

*In our humble opinion, our recommended methodology on precise cell-type annotation for single cell RNA sequencing (scRNA-seq) should be considered from multiple perspectives as* *follows:*

***(A) Avoid potentially false assumption of the cross-species conserved gene expression pattern.*** *Homologous genes across different species may have different expressing patterns. Take HD-ZIP III gene family across angiosperms as an example (please see below for Response Fig. 2), these genes are basically (i) not expressed in Magnoliales nor Trochodendrales (PMID: 36624504), (ii) expressed mainly in vessel element lineage in Myrtales (PMID: 36624504), (iii) expressed in organizer cells in Brassicales (PMID: 30626967), and (iv) respectively expressed highly in organizers in Populus trichocarpa and in ray parenchyma cells and vessel elements in Populus alba var. pyramidalis of Malpighiales (PMID: 36624504). Using HD-ZIP III gene family as organizer marker genes from Arabidopsis thaliana of Brassicales for cell-type annotation in Populus alba var. pyramidalis would unexpectedly annotate non-organizers cells. We summarized the marker genes used in the four studies mentioned in this Correspondence (please see Response table). Chen et al, Li et al and Xie et al all used marker genes based on the assumption of cross-species conserved gene expression pattern.*

***Response Figure 2.*** *Expression patterns of HD-ZIP III gene family across angiosperms. The phylogeny (from left to right) represents Malpighiales (Populus alba var. pyramidalis and Populus trichocarpa), Brassicales (Arabidopsis thaliana), Myrtales (Eucalyptus grandis), Trochodendrales (Trochodendron aralioides) and Magnoliales (Liriodendron chinense). Orange color represents the cell types with HD-ZIP III expression.*

***(B)*** ***Nucleotide sequence-based information are much more reliable.*** *As mentioned above, colorimetric test or fluorescence generate colors or lights as signals for users to define gene expression pattern. The validity of these signals is usually judged subjectively. In contrast, nucleotide sequence-based signals generated by sequencing are much more reliable. The signal specificity can be calculated by sequence alignment, and the transcript abundance can be quantified. Both specificity and quantification are examined rigorously by statistical tools.*

***(C) Use “marker transcriptomes” instead of “marker genes”.*** *In plants, protoplasting appears to be the major method for cell isolation for scRNA-seq. The transcriptomic profiles of plant cells would be perturbed during the procedure of cell isolation. Many scRNA-seq studies used high transcriptomic correlation between bulk tissue (before cell isolation) and the pool of all protoplasts (after isolation) to demonstrate the minimized effects on the perturbation of the transcriptome during cell isolation (PMID: 14671301; PMID: 34809675; PMID: 30913408). However, the comparison between bulk tissue and all protoplasts are based on the averaged transcript abundance. For example, the transcript abundance of gene A are respective 1 and 199 TPM (transcript per million) in xylem cell#1 and cell#2, but are both 100 TPM in their corresponding protoplasts. In this scenario, one would observe very high correlation between bulk xylem cells and the pool of protoplasts, but in reality gene A in the two xylem cells suffers* *gigantic changes on transcript abundance during cell isolation. In other words, it is challenging to identify the gene expression patterns that have changed and those that have remained unchanged during cell isolation.*

*Besides the transcriptomic perturbation during protoplasting, different methods used to reveal gene expression patterns unavoidably introduce additional bias into the interpretation of endogenous gene expression patterns. We take a* *schematic example for the cell-type annotation of scRNA-seq results (**transcriptomic profiles of protoplasts) on libriform fibers and vessel elements using lcmRNA-seq* *(please see below for Response Fig. 3). Both libriform fibers and vessel elements in planta (Response Fig. 3A) encounter transcriptomic changes during scRNA-seq (by protoplasting and sequencing) and lcmRNA-seq (by LCM cell collection and sequencing) (Response Fig. 3A). If only one marker gene is used for cell-type annotation (Gene 6 in Response Fig. 3B), then the cell-type annotation may suffer by the unexpected changes of expressing pattern. Instead, using the whole transcriptomes for cell-type annotation would alleviate the impact of dramatically changes on few genes (Response Fig. 3B).*

*As a result, a robust methodology for cell-type annotation of scRNA-seq results should consider all three criteria above. The ignorance of any of these criteria would bring unexpected bias.*

***Response Figure 3.*** *Schematic example for the cell-type annotation of* *scRNA-seq results. (A) Transcriptomic profiles of xylem, protoplasts and LCM in libriform fiber and vessel elements.* *(B) Cell-type annotation by marker gene or in situ transcriptomes.*

**(2)** The conclusion on Tung et al having the most correct cell annotations and hence xylem trajectories is based on a new analysis of data published in the four papers. In this analysis they correlate the expression of marker genes used in three of the articles to their expression reported in three samples, collected by laser capture microdissection (LCM), in Tung et al.  This analysis is based on the assumption of the authors that the laser dissection method is the ultimate method/proof and the only reliable method to validate cell identities. However, LCM has inherent problems.

*(2-1)* First of all, there is a risk of including unwanted neighbouring cells (or cells below) from the sections during LCM.

*----- [Author Response] -----*

*We thank the recognition of Reviewer#1 on our LCM results mentioned in Major Concern 3-3: “Looking more carefully in the Reviewer files of Tung et al (Additional file 17), it seems that the LCM sampling was done very carefully” “This makes me confident with the LCM data of Tung et al”.*

*To avoid the risk of the collection on unwanted cells, we have conducted the LCM cell collection with extremely careful designs. We categorized the unwanted cells into three types, and explained our designs to avoid the collection of these unwanted cells:*

***(A) Unwanted neighboring cells.*** *We strictly defined the area on cross-sections or tangential-sections to avoid this type of contamination. Detailed information was described in Figure 1 of Tung et al (Fig. 1C-E for libriform fiber collection; Fig. 1G-I for vessel element collection; Fig. K-M for ray parenchyma collection). Tung et al also provided further information for an easier understanding on the definition of collected area, such as schematic 3D structure, cross-section perspective, and tangential-section perspective with their corresponding locations on the stem (Fig. S5 and S6 in Tung et al).*

***(B) Unwanted cells below.*** *The following description was extracted from the Reviewer files of Tung et al (Additional file 17): “During cell collection, we visualized and selected our targeting area, which contained the cell-type of interest. For a sample section, one side can be visualized, and the other side contacted the slide, which cannot be visualized. When laser cut through the selected area on a section, we collected both visualizable and unvisualizable regions. To avoid the collection of unwanted cells, the thickness of sections should contain only one layer of cells, or should be even less than the length or width of one layer of cells, which can thus avoid the unwanted cells hiding in the unvisualizable regions. We set the thickness of cross- and tangential-sections as 16 µm. For cross-sections of libriform fiber collection, the thickness is much less than the cell length of libriform fiber (500-750 µm) (PMID: 17403651). For tangential-sections of vessel element collection, the thickness is also much less than the cell diameter of vessel elements (34-48 µm) (PMID: 17403651).”*

***(C) Unwanted scattered cell fragments.*** *The following description was extracted from the Reviewer files of Tung et al (Additional file 17): “Laser cutting is actually a process of burning out the cells by laser on the selected paths. While burning out the cells, laser cutting also generates tiny fragments. These fragments would either fall down into the collecting caps along with the targeting cell area, or remained on the slides. The fragments remained on the slides may fall down and get collected in the next rounds of cutting. Thus, each round of cutting would result in the collection of targeting cell area and the fragments generated on such round or* *previous rounds. As a result, each slide was only used for the collection on one cell-type to avoid cross contamination of cell types caused by the collection of unwanted residual fragments.”;*

*“To obtain robust and reliable results, all of the criteria had to be strictly fulfilled throughout the cell collection, including the section thickness setting, exclusion of the unwanted residual fragments, and all of the other techniques mentioned in the manuscript. These time-consuming and delicate-labor-intensive tasks led to a gigantic obstacle for the success of transcriptomic comparison among three xylem cell types, which may explain the reason that none of the previous studies accomplish this critical and necessary goal.”*

*(2-2)* Trials by others have failed to provide RNA from the intended cell types due to production of heat by the laser (e.g. Larisch et al 2012 Plant Physiology 160: 1515-1529), which is many times overcome by increasing the size of captured area (and losing the cell specificity).

*----- [Author Response] -----*

*The comparison of LCM procedure between Tung et al and Larisch et al (please see below for Response Fig. 4) may explain their differences.*

***(A) Sample preparation.*** *Tung et al used fresh tissue for stem sectioning by cryostat, and Larisch et al used paraffin-embedded tissue for sectioning by microtome. Paraffin embedded tissue sectioning requires tremendous longer processing time, and tissues were incubated under many different working temperatures that may be unfavorable for RNA integrity, such as room temperature (RT), 30°C, 42°C and 56°C. In contrast, fresh tissue sectioning by cryostat could ensure the whole process conducted at -20°C, which could minimize the risk of RNA degradation.*

***Response Figure 4.*** *The LCM procedure of* *Tung et al* *(PMID: 36624504) and Larisch et al (PMID: 22992511). LN, liquid nitrogen. NA, not available.*

***(B) Platform for transcriptomic analyses.*** *Larisch et al used microarray for transcriptomic analyses, which generally requires 10-100 µg RNA as input (PMID: 15119961). Tung et al* *used RNA sequencing, which requires less than 1 µg RNA as input. Namely, the transcriptomic analyzing platform used in Tung et al needs much less RNA input.*

***(C) Amount of collected cells.*** *Larisch et al collected 80 pieces of cells, but the area of each piece was not mentioned. Through our comparison of Figure 2A of Larisch et al and Figure 1K of Tung et al, the piece area in these two articles is similar. Tung et al collected 1,600,000 μm^2^ area for each cell type. Take ray parenchyma cells for example, the area of the majority of collected pieces is around 500-2500 μm^2^, so at least 640 pieces (1,600,000/2500=640) were collected by Tung et al.*

*Tung et al conducted sample preparation in a much shorter processing time under more favorable temperature, used the platform requiring much less RNA input, and collected much more samples. These reasons could explain the better LCM quality in Tung et al.*

*(2-3)* It is also difficult with the LCM technique to cover exactly the same area as obtained with the SDX protoplasting. Tung et al includes cells for LCM from 20 cell layers of cryosections, while the schematic figure 1A in Tung et al indicates that SDX protoplasts were derived from eight cell layers.

*----- [Author Response] -----*

*As mentioned by Reviewer#1, it is really difficult for LCM to cover exactly the same area as protoplasting. The LCM results in Tung et al were obtained from 20 outermost cell layers of the debarked stem, which already required more than 1 year of processing time to collect three cell types. Cells from protoplasting are from 6 outermost cell layers of the debarked stem. The difficulty for 6-layer cell collection using LCM would increase tremendously, and the increased difficulty may require at least more than 5-year processing time, which appears not quite practical.*

*The LCM results in Tung et al could identify specific cell clusters from scRNA-seq, and the identified/annotated cell clusters showed consistent characteristics with previous studies. For example, secondary cell wall (SCW) biosynthetic genes were heavily expressed in libriform fibers, which possess the thickest SCW among three cell types. The expansin with highest transcript abundance was specifically expressed in vessel elements, which require highest extent of cell expansion among three cell types. Many photosynthetic genes were specifically expressed in ray parenchyma cells, which supported previous studies that chlorophyll-containing plastids in ray parenchyma cells can carry out woody tissue photosynthesis.*

*(2-4)* Furthermore, it is difficult if not impossible, to define in the cryosections whether the fast-differentiating cells, such as vessel elements, are viable.

*----- [Author Response] -----*

*The previous study has demonstrated that vessel elements are viable within 400 µm from cambium in Populus (PMID: 19175765). The LCM collected vessel elements in Tung et al were all within 400 µm.*

**(3)** In Tung et al, it seems from the data analysis in figure 1J that the LCM vessel sample represents transcription of genes that can be found in several scRNAseq cell clusters.

*(3-1)* Does it mean that the LCM sample represents several cell types or perhaps both fibers and vessels at different developmental stages, or does it mean that vessel elements (at different stages of development) are present in several clusters in the scRNAseq data?

*----- [Author Response] -----*

*Cell development is a continuous process, and the corresponding transcriptomic changes during the development are also continuous. The cell cluster with the highest* *correlation to the marker transcriptome of vessel elements would be annotated as vessel elements. In* *Figure S7B in Tung et al (pasted below as Response Fig. 5A), the marker transcriptome of vessel elements showed the highest correlation with Ptr1 (blue), and Ptr1 was thus annotated as vessel elements. We here also provided* *quantitative results as a box plot with statistical analyses of the data* *(Response Fig. 5B).* *Comparing each cluster from Ptr2-Ptr10 to Ptr1, the p-values are all significant (p-value < 2.2e-16).*

*Several cell clusters show relatively high correlation with the marker transcriptome of vessel elements, because these cell clusters are precursor cells or the cells shared common precursor cells with vessel elements. For example, Ptr2 (brown), as the intermediate precursor cells of vessel elements, showed the second highest correlation with the marker transcriptome of vessel elements (Response Fig. 5C). The earlier cells (early precursor cells [Ptr4 in green]) and even earlier cells (organizer cells [Ptr6 in purple]) showed a further decreasing trend of correlation (Response Fig. 5C).*

***Response Figure 5.*** *Correlation analyses between scRNA-seq and lcmRNA-seq of vessel elements from Tung et al (PMID: 36624504). (A) Data from Figure S7B* *of Tung et al. (B) The box plot reanalysis of the same data from Figure S7B of Tung et al. Comparing each cluster to Ptr1 (Blue), the p-values are all less than 2.2e-16. (C) The box plot reanalysis of the Ptr1 (vessel elements, blue), Ptr2 (intermediate precursor, brown), Ptr4 (early precursors, green) and Ptr6 (organizer, purple) selected from Figure S7B of Tung et al.*

*(3-2)* Fig. S7 in Tung et al also shows that there is no correlation between gene expression in the LCM vessel sample with any of the scRNAseq clusters, rendering the true identity of the vessel LCM and/or the annotation of the clusters unclear.

*----- [Author Response] -----*

*Although the overall correlation of cell clusters from scRNA-seq is low with the LCM vessel* *elements, we still could observe many cells with obvious relative high correlation around 0.4 (please see Response Fig. 5A above).*

*For the cell-type annotation of single cell transcriptomes of xylem protoplasts, we used correlation analyses between lcmRNA-seq and scRNA-seq. These two platforms (scRNA-seq and lcmRNA-seq) started from SDX followed by protoplasting and laser cutting, respectively. As we mentioned above in Major Concern 1-2 (please see Response Fig. 3 above), these two platforms lead to different transcriptome perturbation during the experimental procedure, which caused such relative low correlation between these two platforms. However, obvious correlation difference could still be observed among different cell types. For example, we still could observe a group of highest correlated cells. In other words, even after vessel elements undergo protoplasting and laser cutting, they continue to exhibit the highest correlation with each other, rather than with other cell types such as libriform fibers or ray parenchyma cells.*

*(3-3)* Looking more carefully in the Reviewer files of Tung et al (Additional file 17), it seems that the LCM sampling was done very carefully, and that the gene expression results from their LCM data on vessels and fibers correspond very well to previously published RNAseq data on laser microdissected fibers and vessels. This makes me confident with the LCM data of Tung et al but at the same time questions the correct interpretation of their scRNAseq clustering.

*----- [Author Response] -----*

*We greatly appreciate Reviewer#1’s recognition of our LCM platform.*

*(3-4)* It says in the Additional file 17 in Tung et al for instance that "In this study, XCP2 is enriched in libriform fibers in the scRNAseq results, but is enriched in vessel elements in the lcmRNAseq results". It would be really interesting for the community working with this technology to know what could be the reason(s) behind this, especially as XCP2 is very commonly used as a marker gene in scRNAseq analyses. My guess is that the cell and tissue composition in the laser microdissected samples and in the protoplast population is different. For instance, it is likely that differentiating vessel elements are poorly represented in the scRNAseq data due to their large size that is incompatibility with both the protoplasting method and the chromium technology, which is why vessels expressing XCP2 are not enriched in cluster 1 in Tung et al. Why XCP2 expressing fibers are not enriched in the LCM fiber sample, is more difficult to explain. Any comments on all these topics?

*----- [Author Response] -----*

*As we mentioned in the response to Major Concern 1-2: “different methods used to reveal gene expression patterns unavoidably introduce additional bias into the interpretation of endogenous gene expression patterns.” Such bias could possibly happen in the way mentioned by Reviewer#1. Although LCM in situ transcriptomic analyses appear to be the most reliable platform to collect information for cell-type annotation, this platform unavoidably introduces certain levels of bias into the gene expression pattern on certain genes (please see Response Fig. 3A above). We thus emphasized the necessity of using “marker transcriptomes” as a whole for cell-type annotation instead of using selected “marker genes”.*

**(4)** The commentary makes several unmotivated claims on misinterpretation of the data in Chen et al.

*(4-1)* First, it is claimed on lines 87-89 that Chen et al misinterpreted the effect of debarking. This is however not the case. They show in supplementary figure S1A very clear microscopy images on the breaking point after peeling of the bark, which does not correspond to what is drawn in Figure 1F and G in the current manuscript. In Chen et al, the breaking point is not important either, and does not influence the conclusions since scRNAseq data from the bark and the secondary xylem were pooled for the data analysis. Please correct (and redraw in figure 1) this part concerning Chen et al.

*----- [Author Response] -----*

*According to Reviewer#1’s suggestion, we revised the schematic figures of “Chen et al” in Fig. 1F and 1G based on their anatomical results (Fig. S1A in Chen et al) and cell-type annotation (Fig. S4 and S5 in Chen et al). We extracted the information of Fig. S1A, S1D, S4, and S5 from Chen et al, and re-assembled a figure below on the basis of Chen et al’s data for the ease of understanding (**Response Fig. 6).*

*From* *the anatomical results of Chen et al, the debarking effect was shown as the separation of cambium and differentiating xylem (Response Fig. 6A). The vast majority of cambium cells were remained on the “bark” part (indicated by the green dashed region) (Response Fig. 6A). On the “wood” part, the vast majority are differentiating xylem cells.*

*In the cell-type annotation of Chen et al (Response Fig. 6B and 6C), cell clusters 1, 2, and 10 were annotated as cambium cells, and these three cell clusters were composed of 1671 cells from “wood” part (816+686+169) and 113 cells from “bark” part (24+81+8) (Response Fig. 6D). The vast majority of annotated cambium cells located on the “wood” part.*

*Namely, in Chen et al, the vast majority of cambium cells located on the “bark” part based on the anatomical results but on the “wood” part using the cell-type annotation results. Thus, the anatomical analyses and cell-type annotation were conflicted in Chen et al. Instead of claiming the misinterpretation of the data, we revised the schematic figures of “Chen et al” in Fig. 1F and 1G to demonstrate their conflicting results.*

***Response Figure 6.*** *The anatomical debarking results and cambium cell annotation results from Chen et al (PMID: 34809675). (A) The anatomical results were extracted from Fig S1A of Chen et al. The green dashed region indicates the cambium. (B) UMAP visualization of cells from bark and wood parts from Fig S4 of Chen et al. (C) UMAP visualization of 20 cell clusters from Fig 1B of Chen et al. The cluster 1, 2 and 10 were annotated as the cambium region. (D) The results extracted from Fig S5 of Chen et al. The cell numbers of “bark” and “wood” parts in cluster 1, 2 and 10 were shown.*

*(4-2)* Second, it is claimed on lines 96-97 on the basis of the correlation analysis in figure 1I that Chen et al annotated "vessel elements/libriform fibers unexpectedly as libriform fibers".  I cannot however find anywhere in Chen et al. information on any marker genes specifically for fibers and vessels. They reported throughout the manuscript results on only "xylem elements" since obviously they did not find evidence in separating the developmental trajectories for fibers and vessels. So it is not surprising that their xylem marker genes (that were not claimed to be specific to fibers or vessels) correlated with the localisation of the LCM sample of fibers. Apparently the markers they used were mostly expressed in the fibers. This needs to clarified and correctly reported in the commentary, and corrected in figure 1I.

*----- [Author Response] -----*

*We agree that the marker genes used in Chen et al annotated XSCW (xylem cell with SCW) instead of vessel elements or libriform fibers. Based on the quoted sentence from Chen et al: “These results imply that the XSCW branch is composed of at least two cell types, vessels and* *fibers.”, we revised our description in our revised manuscript as following: “**As a result, the marker genes used from both studies (Li et al for vessel elements; Chen et al for xylem cells composed of at least vessel elements and libriform fibers) unexpectedly annotated libriform fibers (Fig. 1I).” Please see lines 106-108 in the revised manuscript.*

*(4-3)* Third, it is claimed in lines 107-109 that annotation of vascular cambium and ray parenchyma cells were misplaced in Chen et al. The evidence for this claim does not become clear from Tung et al, and should be clarified here thoroughly.

*----- [Author Response] -----*

*We concluded the misplacement of cambium and ray parenchyma cells based on (A) the incorrect annotation on cambium cells of Chen et al, and (B) the analyzed results of the reannotation of the incorrectly annotated cambium.*

***(A) Incorrect cambium cell annotation.*** *As we mentioned in the response to Major Comments 4-1, the vast majority (~94%, 1671/[1671+113]=93.67%) of cambium cells were annotated in the “wood” part in Chen et al. The major strategy in Chen et al for the annotation on cambium cells for the developmental lineage construction was based on the gene expression pattern of HD-ZIP III genes. Chen et al asserted that HD-ZIP III genes were prominently expressed in cambium cells. The following sentence was quoted from Chen et al: “We found that multiple HD-ZIP III transcription factor genes (such as PtrHB4, PtrHB7, and PtrHB8, all of which play crucial roles in regulation of vascular cambium activity and xylem development in poplars [63, 64, 82]) were prominently expressed at the beginning of pseudo-time.” Chen et al marked the beginning of pseudo-time as cambium region. We pasted Fig. 3A and 3B of Chen et al below for easier understanding (Response Fig. 7). However, among these three references “63, 64, 82” cited in this sentence, none of these references reported the prominent expression of PtrHB4, PtrHB7, nor PtrHB8 in cambium.*

***Response Figure 7.*** *The results from Fig 3A and 3B of Chen et al (PMID: 34809675).*

*First, reference 63 (PMID: 28905477): We pasted Figure 1 from this article below. PtrHB4 was expressed in many different tissues, including shoot apical meristem (SAM), primary xylem, phloem, and interfascicular parenchyma cells (Figure 1g-i below). SAM, not cambium, is the tissue with highest expression of PtrHB4 (Figure 1a below). Thus, this reference does not provide evidence on the prominent expression of HD-ZIP III in cambium.*

Figure legends from Figure1:

Expression pattern of *PtrHB4* during vascular cambium formation.

(a) Expression of *PtrHB4* in *Populus* stem analysed by RT-qPCR. *PtrActin1* was used as a reference gene. Bars are means ± SD of n = 3 biological replicates.

(b) Histochemical analysis of GUS activity in shoot tip of *PtrHB4pro: GUS* transgenic plants.

(c-j) Immunolocalization analysis of *PtrHB4* in shoot apex (c), in IN1 (d and e, indicating continual sections from top to bottom), (f) magnification of the framed section in (e). In IN2 (g), (h) magnification of the framed section in (g), (i) magnification of the framed section in (h) and in IN12 (j). IN, internode; SAM, shoot apical meristem; pca, procambium; ca, cambium; fca, fascicular cambium; ica, interfascicular cambium; ipc, interfascicular parenchyma cells; pvb, primary vascular bundle; pph, primary phloem; pxy, primary xylem; sph, secondary phloem; sxy, secondary xylem; spf, secondary phloem fibre. Bars: 1 mm in (b), 200 μm in (c), (d), (e) and (g), 100 μm in (f), (h) and (j), 20 μm in (i).

*Second, reference 64 (PMID: 23288865): We pasted Figure 2 from the article below, and added four red arrows. PtrHB7 and PtrHB8 were highly expressed in both cambium and xylem.*

*Third, reference 82 (PMID: 23647338): We pasted a part of Figure 8 from the article below, and added three red arrows. The transcript abundance of PttHB8 in wild type was used for the comparison to that in transgenics. The absolute transcript abundance of PttHB8 in wild type was not reported in this study. Thus, this study did not provide the information on the prominent expression of PttHB8 in cambium.*

*Thus, using the expression pattern of HD-ZIP III family for cambium annotation in Populus would unexpectedly annotate other cell types into cambium.*

***(B) Re-annotation of the “annotated cambium”.*** *Since the “annotated cambium” in Chen et al may not represent cambium, then what cell types do these “annotated cambium cells” belong to? In Fig. S36 and S37 of Tung et al, the scRNA-seq results from the “wood” part of Chen et al were integrated with other four species across angiosperms for cell-type annotation and developmental lineage construction. The developmental lineage of the “wood” part in Chen et al started from fusiform organizer cells and ray organizer cells to vessel elements/libriform fibers and ray parenchyma cells, respectively. We here provided the quantitative analyses of the transcript abundance of HD-ZIP III genes (Response Fig. 8), which was significantly higher in the more differentiated cells, such as ray parenchyma cells, than that in each type of organizer cells (Response Fig. 8A) or the average of both organizer cells (Response Fig. 8B). Thus, the cambium annotation in Chen et al appears to be incorrect.*

***Response Figure 8.*** *The transcript abundance of HD-ZIP III genes in ray and organizer cells from reannotated data of Chen et al. (A) The normalized UMI of HD-ZIP III genes in ray organizer (orange), fusiform organizer (purple) and ray parenchyma (pink) cells. (B) The normalized UMI of HD-ZIP III genes in organizer (the average of ray organizer and fusiform organizer) and ray parenchyma cells.*

*To focus on the mis-annotation of cambium cells of Chen et al, we now revise the original sentence, and it reads as: “**, and their cambium annotation appears to be incorrect due to the false assumption of HD-ZIP III as the rightful marker genes.” Please see lines 118-120 in the revised manuscript.*

Minor Comments

**(1)** A correlation analysis between marker genes in Tung et al (for instance marker genes shown in Sfigure21 and 22) and the LCM data needs to be added to figure 1I, similar to what is shown for the other three papers.

*----- [Author Response] -----*

*We have added the correlation analysis of the marker genes in Tung et al to Figure 1I. These marker genes, selected from in situ transcriptomic analyses, could specifically annotate each cell type.*

**(2)** Since one the aims with the commentary is to put focus on correct annotation of the different xylem cell types and correct anatomical knowledge, it is important the authors adhere to well established nomenclature on the cell types and their differentiation in the cambial zone (according to the references Larson 2012 and Evert 2006).

*(2-1)* Lines 69-72. The term "fusiform system" is artificial, and definitely not widely, if at all, used. Vascular cambium contains fusiform initials that are named "fusiform" due to the origin of the word (shape that is broad in the middle and narrows down towards the tip). I strongly discourage inferring terms to the literature that are confusing and that don't correspond to the etymology of the word "fusiform".

*----- [Author Response] -----*

*We thank the suggestion of Reviewer#1, and revised “fusiform system” into “**axial system”.*

*(2-2)* Furthermore, vascular cambium does not differentiate or grow in any direction, but produces xylem precursor cells inwards in the stem.  Cells don't either differentiate into stem differentiating xylem (SDX) but into xylem fibers, vessels or parenchyma.  Please reformulate this part.

*----- [Author Response] -----*

*We revised the sentence and it now reads as: “stem cells (initials) proliferate into proximal cambium to produce differentiating xylem with two architectural systems, axial system (growing upward and downward) and radial system (growing inward and outward).” Please see lines 72-74 in the revised manuscript.*

*(2-3)* The use of the term "SDX" is not justifiable in this context. Only two of the papers uses this term. Please use instead the more simple term "differentiating xylem".

*----- [Author Response] -----*

*We revised “stem differentiating xylem (SDX)” into “differentiating xylem”.*

**(3)** it is discussed in lines 79-85 that the debarking separates vascular cambium and "SDX", and that it is very consistent among different species grown in various regions. This is an oversimplification since cambial cell divisions rates influence the breaking point in the cambial region when debarking. But clearly the most fragile area is the expanding xylem elements, resulting in a rupture in this tissue during debarking. However, it cannot be excluded that cambial cells sometimes rip off along with the secondary xylem tissues. This is supported by frequently observed expression of not only the cambial markers but also genes related to cell division related and even phloem precursor identity. Therefore, talking about SDX as if it was always reproducibly collected and/or well defined is misleading. The authors are however completely correct in that it is important (and helpful for the data interpretation) to know where the debarking occurs. However, I would like to point out that an image on a 300 um wide cross section (as in Li et al.) or schematic pictures (as in Tung et al.) are not enough to demonstrate the fracture site and do not allow excluding for instance cambial cells ripping off together with the differentiating xylem.

*----- [Author Response] -----*

*We agree with Reviewer#1 that we may not be able to conclude the exact break point of debarking by these anatomical results. We also agree that differentiating xylem on the surface of debarked stem may not be always well defined due to the uncertainty on the break point of debarking. For example, the surface of debarked stem may contain few residual cambium cells, or few differentiating xylem cells were ripped off together with cambium.*

*We thank the recognition of Reviewer#1 that “The authors are however completely correct in that it is important (and helpful for the data interpretation) to know where the debarking occurs.” The anatomical information is critical for the data interpretation of scRNA-seq results. Although the exact break point of debarking could not be concluded, the effect of debarking was highly consistent among many studies published in high-impact journals (top 5%), which could provide valuable information on the cell-type annotation and lineage construction.*

*Through the anatomical analyses, these studies all concluded the debarking effect as the separation of bark (containing cambium) and differentiating xylem:*

*(i) Lin et al., 2014 Nature Protocols (2.60% [2/77, Biochemical Research Methods]).*

*(ii) Li et al., 2021 Journal of Integrative Plant Biology (2.94% [7/238, Plant Science]).*

*(iii) Chen et al., 2021 Genome Biology (2.92% [5/171, Genetics & Heredity]).*

*(iv) Tung et al., 2023 Genome Biology (2.92% [5/171, Genetics & Heredity]).*

*Their anatomical results on debarking were summarized in the “Reviewer#3 minor details 1 of Reviewer files in Tung et al (Additional file 17)”. We pasted the summarized figure below for easier understanding (Tung et al was shown as “This study”).*

*Another study also described the debarking effect as the separation of bark (containing cambium) and differentiating xylem, and was published in a top 10% journal.*

*(v) Bossinger and Spokevicius, 2018 Journal of Experimental Botany (7.56% [18/238, Plant Science]).*

*With these highly consistent anatomical results among the five studies, it should be reasonable to describe that debarking separates cambium and differentiating xylem.*

*We thus revised and toned down our description:*

*(A) We removed the sentence: “After debarking, only SDX remains on the surface of the stem”.*

*(B) We toned down the sentence “the subsequent protoplast isolation then allows the collection of pure SDX protoplasts without the contamination of phloem or cambium cells (Fig. 1E) [15, 17, 19, 20].” It now reads as: “**The subsequent protoplast isolation then allows the collection of* *differentiating xylem protoplasts (Fig. 1E) [15, 17, 19, 20].” Please see lines 84-86 in the revised manuscript.*

*(C) With other revisions, the original sentences in lines 79-85 now read as: “**The first step of such protoplasting pipeline is stem debarking. Anatomical analyses showed an obvious separation of vascular cambium and differentiating xylem after debarking, and the bark contains phloem and vascular cambium (Fig. 1E). The subsequent protoplast isolation then allows the collection of differentiating xylem protoplasts (Fig. 1E) [15, 17, 19, 20]. Such anatomical results after debarking are very consistent among different species grown in various regions, such as North America, East Asia and Australia [15, 17, 19, 20].” Please see lines 81-88 in the revised manuscript.*

**(4)** Lines 97-91. Chen did not misinterpret the anatomy of debarked stem. Xie et al. does not give information in the paper on where the bark is detached, and it is difficult to see how their misinterpretation could have led to "subsequent mis-annotation of SDX cells". They define a phloem cluster, and without looking at the details of this cluster, I would not disclose the possibility that this cluster is not composed of phloem precursor cells. This is important for the reader to know. Is it certain that the phloem cluster in Xie et al is not correct, as claimed on lines 90-91? If no better evidence is provided on misintepretation in the cell composition of the sample (influenced by the fracture site), please remove the focus on the sampling method in figure 1.

*----- [Author Response] -----*

*We revised the schematic figure of Xie et al Figure 1F based on Reviewer#1’s suggestion. As mentioned by Reviewer#1, Xie et al did not provide anatomical information on debarking, we revised this part as “not available”.*

*Although Xie et al did not provide anatomical results on debarking, the following three reasons could justify the similar debarking effects among all four studies described in this Correspondence, including Xie et al.*

*(A) All of these four studies used Populus debarked stem for protoplasting.*

*(B) All of these four studies used the same protoplasting platform reported in Lin et al 2014 (PMID: 25144270). None of these studies reported any revisions on the protocol.*

*(C) The anatomical results of debarking effect are consistent among Tung et al, Chen et al, and Li et al.*

*(D)* *The overlapping rates of the scRNA-seq results using the protoplasts isolated from debarked stem are extremely high (Tung and Chen “wood” = 96.7%; Tung and Li = 99.5%; Tung and Xie = 99.9%) (Response Fig. 9).*

***Response Figure 9.*** *The overlapping rates of the scRNA-seq results using the protoplasts isolated from debarked stem. (A) Tung et al and Chen et al. (B) Tung et al and Li et al. (C) Tung et al and Xie et al.*

*Without direct evidence on the break point of debarking, we toned down our description, and the sentences now read as: “**Among the four studies, Tung et al, Chen et al and Li et al used anatomical analyses to ensure the debarking effect as the separation of cambium and differentiating xylem (Fig. 1F). Chen et al reported contradicting results between their anatomical analyses and cell-type annotation. The vast majority of cambium cells located on the bark side based on the anatomical results but on the debarked stem using the cell-type annotation results (Fig. 1F, 1G). The anatomical analyses of debarking effect were not reported in Xie et al (Fig. 1F). Upon the use of same protoplasting pipeline, the scRNA-seq results between Xie et al and Tung et al showed extremely high overlapping rates (99.9%, see Response to Reviewer#1), which demonstrates the highly similar debarking effects between these two studies. The cell-type annotation of Xie et al on debarked stem included cambium and phloem, and conflicted to the suggested debarking effect (Fig. 1F, 1G). The contradicting results between anatomy and cell-type annotation very likely led to subsequent mis-annotation of differentiating xylem cells into phloem and vascular cambium.” Please see lines 88-101 in the revised manuscript.*

**(5)** One more paper has been published since the submission of this commentary. Please include descriptions and analysis of this manuscript as well.

*----- [Author Response] -----*

*Two related articles were published since the submission of this Correspondence.*

*(A) Yu et al. Title: Integrated mass spectrometry imaging and single-cell transcriptome atlas strategies provide novel insights into taxoid biosynthesis and transport in Taxus mairei stems. (PMID: 37219365)*

*The targeted species in this article is Taxus mairei, which is a species in gymnosperms. All species discussed in this Correspondence are angiosperms. Since xylem cell types in gymnosperms are different from that in angiosperms, the developmental lineages reported in Yu et al is not comparable to that in this Correspondence.*

*(B) Du et al. Title: High-resolution anatomical and spatial transcriptome analyses reveal two* *types of meristematic cell pools within the secondary vascular tissue of poplar stem. (PMID: 36895162)*

*In this article, spatiotemporal sequencing (10X Genomics) was used to explore stem development in poplar. They reported a new developmental model throughout vascular primary growth to secondary growth. Their research focus was not on the detailed xylem development. In addition, current resolution of spatiotemporal sequencing generated by 10X Genomics (product name as Visium) is 55-μm in diameter of each spot. Such spots are larger than the largest cell type in xylem, vessel elements (34-48 µm). Namely, each spot would contain many cells, even many cell types. Thus, the resolution of such spatiotemporal sequencing platform may not be suitable to dissect cell-level development of differentiating xylem, but is an excellent tool to demonstrate tissue-level development, such as primary and secondary growth of vasculature. As a result, the results reported in this article are also not comparable to that in this Correspondence.*

*We thus prefer not to include these two articles in this Correspondence.*

**(6)** The English language should be checked. For instance lines 46-48, 51-54, and 56.

*----- [Author Response] -----*

*We have revised these sentences. Please see lines 47-49, 53-56 and 58-60 in the revised manuscript.*

**Reviewer #2**

The power of single-cell technologies in plant biology research has been remarkable, with applications expanding beyond Arabidopsis to other plant species. However, a standardized paradigm for the application of this new technology is yet to be established. In this correspondence, valuable insights are presented for current single-cell studies in plant developmental research. Integrating in situ transcriptomics and anatomical information in single-cell analyses holds great promise for precise cell annotations. Using a previous study as an example, the authors compare key findings on xylem development with three other single-cell studies. Through this comparison, they highlight potential mis-annotations of cell types in previous studies and emphasize the necessity of combining in situ transcriptomics and anatomical information in single-cell analyses. Overall, the manuscript is well-written and the presents a thought-provoking perspective.

*----- [Author Response] -----*

*We are thankful for the Reviewer#2's positive feedback and kind acknowledgment of our work.*

**Second round of review**

**Reviewer 1**

This reviewer thanks the authors of taking into consideration many of the concerns raised towards the initial submission of the commentary by Chen et al. Interesting details and data is included in the response to the reviewers letter. I am still somewhat concerned about the handling of some of the results from one of the papers commented on in this manuscript.

1. On lines 90-93, the authors state "Chen et al reported contradicting results between their anatomical analyses and cell type annotation. The vast majority of cambium cells located on the bark side based on the anatomical results but on the debarked stem using the cell type annotation results".

Cell type annotation (on the basis of gene expression) can be misleading for several reasons. In this case, the fact that the cells from the bark side end up in the cambium clusters in Chen et al. could be due to problems in cell type annotations but also due to i) cambial cells ripping off to the wood side while peeling the bark, or ii) the use of the term "cambium".

i) The authors refer in their response to several papers where the fracture site after peeling of the bark has been shown to reside between the cambium and the differentiating xylem. However, it has been frequently shown that the tissues scraped from the exposed wood express well-established markers of cambial cells (or initials) and even markers of early phloem identity, which shows that the fracture site is perhaps not always exactly in the same place throughout the circumference of the stem and that cells from the whole cambial region can end up in the exposed wood side. Even Tung et al. seems to have cells from the cambial region on the basis of the enrichment of genes from the meristem related GO term (Figure S14). Unfortunately, the data from Tung et al. is available only as raw data, and hence not easily accessible, but the extended data figures show some expression of for instance ANT, a well-known marker for the cambial stem cells, in the tissues from the surface of the wood.

ii) Chen et al. defines the clusters 1, 2, and 10 as "cambium region" , and it becomes clear from the paper that at least the cluster 2 contains mainly cells that are already specified as xylem elements. It is therefore not surprising that Cluster 2, annotated as "cambium region", is mainly composed of cells from the wood side. The question here is perhaps more about what is considered with the term "cambium region", rather than any major conflicting or controversial results.

For these reasons, I would like to see that these sentences on the lines 90-93 would be written for instance in the following way: In Chen et al, the cambium was located on the bark side based on the anatomical results but vast majority of the cells in the "cambium region" clusters resided on the debarked stem using the cell type annotation results, which demonstrates the challenges in cell type annotation on the basis of the scRNAseq data.

2. Lines 99-101. The sentence is not correct in respect to Chen et al. The "cambium region" clusters are never claimed to consist purely of cambial cells or vascular cambium. I would like to emphasize here rather the importance of careful anatomical inspection of the samples, but also the advantages in this system. Separation of the tissues by peeling, followed by careful analysis if the fracture site, gives important information on the cell types that can be present in the scRNAseq data. This is not possible in Arabidopsis stem.

3. Lines 118-120. The comment on the Chen et al. is not correct. The markers used in Chen et al. to identify the clusters corresponding to the "cambium region" included WOX4, PXY, MP, ANT and CLE47. Chen et al. further reports that "three HD ZIP III transcription factor genes, …, were highly expressed in cluster 5". Cluster 5 is defined as "xylem mother cells". The HD-ZIP III genes are therefore not used to identify the clusters of the cambium region.

In the pseudotime analysis of Chen et al, the HD Zip III family members are indeed highly expressed in cells already in the beginning of the xylem trajectory, but this is not surprising considering the wide expression pattern of these genes. In the AspWood database (plantgenie.org), all HD Zip III family members are expressed in the cambium and coregulated with the cambial marker WOX4. Furthermore, Tung et al. reports the expression of HD Zip III family members in both ray and fusiform "organisers" (figure S13), which altogether suggests that the starting point of the xylem trajectory in Chen et al. includes ray and fusiform initials, and perhaps also cambial stem cells. Taken together, there are uncertainties in the cell annotations in Chen et al, but not due to the expression of the HD ZIP III transcription factor genes.

I would therefore urge the authors to remove the wording "and their cambium annotation appears to be incorrect due to the misuse of HD-ZIP III as the marker genes".

**Authors Response**

**Point-by-point responses to the reviewers’ comments:**

Reviewer #1: This reviewer thanks the authors of taking into consideration many of the concerns raised towards the initial submission of the commentary by Chen et al. Interesting details and data is included in the response to the reviewers letter. I am still somewhat concerned about the handling of some of the results from one of the papers commented on in this manuscript.

*----- [Author Response] -----*

*We agree with all of the three comments raised by Reviewer#1, and thank Reviewer#1 for providing valuable information and perspectives. Accordingly, we have revised our manuscript based on the suggestions. Please see the following for our point-by-point responses to all comments and suggestions.*

1. On lines 90-93, the authors state "Chen et al reported contradicting results between their anatomical analyses and cell type annotation. The vast majority of cambium cells located on the bark side based on the anatomical results but on the debarked stem using the cell type annotation results".

Cell type annotation (on the basis of gene expression) can be misleading for several reasons. In this case, the fact that the cells from the bark side end up in the cambium clusters in Chen et al. could be due to problems in cell type annotations but also due to i) cambial cells ripping off to the wood side while peeling the bark, or ii) the use of the term "cambium".

i)	The authors refer in their response to several papers where the fracture site after peeling of the bark has been shown to reside between the cambium and the differentiating xylem. However, it has been frequently shown that the tissues scraped from the exposed wood express well-established markers of cambial cells (or initials) and even markers of early phloem identity, which shows that the fracture site is perhaps not always exactly in the same place throughout the circumference of the stem and that cells from the whole cambial region can end up in the exposed wood side. Even Tung et al. seems to have cells from the cambial region on the basis of the enrichment of genes from the meristem related GO term (Figure S14). Unfortunately, the data from Tung et al. is available only as raw data, and hence not easily accessible, but the extended data figures show some expression of for instance ANT, a well-known marker for the cambial stem cells, in the tissues from the surface of the wood.

ii)	Chen et al. defines the clusters 1, 2, and 10 as "cambium region" , and it becomes clear from the paper that at least the cluster 2 contains mainly cells that are already specified as xylem elements. It is therefore not surprising that Cluster 2, annotated as "cambium region", is mainly composed of cells from the wood side. The question here is perhaps more about what is considered with the term "cambium region", rather than any major conflicting or controversial results.

For these reasons, I would like to see that these sentences on the lines 90-93 would be written for instance in the following way: In Chen et al, the cambium was located on the bark side based on the anatomical results but vast majority of the cells in the "cambium region" clusters resided on the debarked stem using the cell type annotation results, which demonstrates the challenges in cell type annotation on the basis of the scRNAseq data.

*----- [Author Response] -----*

*We have revised our manuscript using the description provided by Reviewer#1. A part of the revised manuscript was shown below for the ease of reading, and the revised sentences were highlighted by underlines: “Among the four studies, Tung et al, Chen et al and Li et al used* *anatomical analyses to ensure the debarking effect as the separation of cambium and differentiating xylem (Fig. 1F).* *In Chen et al, the cambium was located on the bark side based on the anatomical results, but vast majority of the cells in the "cambium region" clusters resided on the debarked stem using the cell type annotation results, showing the challenges in cell-type annotation on the basis of the scRNA-seq data.” Please see Lines 90-93 in the revised manuscript.*

2. Lines 99-101. The sentence is not correct in respect to Chen et al. The "cambium region" clusters are never claimed to consist purely of cambial cells or vascular cambium. I would like to emphasize here rather the importance of careful anatomical inspection of the samples, but also the advantages in this system. Separation of the tissues by peeling, followed by careful analysis if the fracture site, gives important information on the cell types that can be present in the scRNAseq data. This is not possible in Arabidopsis stem.

*----- [Author Response] -----*

*We have revised the manuscript based on Reviewer#1’s suggestion, and it now reads: “**These results highlighted the importance of careful anatomical inspection of the samples, in which the separation of tissues by peeling provides critical information on the cell types that can be present in the scRNA-seq data.” Please see Lines 99-102 in the revised manuscript.*

3. Lines 118-120. The comment on the Chen et al. is not correct. The markers used in Chen et al. to identify the clusters corresponding to the "cambium region" included WOX4, PXY, MP, ANT and CLE47. Chen et al. further reports that "three HD ZIP III transcription factor genes, …, were highly expressed in cluster 5". Cluster 5 is defined as "xylem mother cells". The HD-ZIP III genes are therefore not used to identify the clusters of the cambium region.

In the pseudotime analysis of Chen et al, the HD Zip III family members are indeed highly expressed in cells already in the beginning of the xylem trajectory, but this is not surprising considering the wide expression pattern of these genes. In the AspWood database (plantgenie.org), all HD Zip III family members are expressed in the cambium and coregulated with the cambial marker WOX4. Furthermore, Tung et al. reports the expression of HD Zip III family members in both ray and fusiform "organisers" (figure S13), which altogether suggests that the starting point of the xylem trajectory in Chen et al. includes ray and fusiform initials, and perhaps also cambial stem cells. Taken together, there are uncertainties in the cell annotations in Chen et al, but not due to the expression of the HD ZIP III transcription factor genes.

I would therefore urge the authors to remove the wording "and their cambium annotation appears to be incorrect due to the misuse of HD-ZIP III as the marker genes".

*----- [Author Response] -----*

*Based on Reviewer#1’s suggestion, we have removed the wording in the revised manuscript.*

**Third round of review**

**Reviewer 1**

I appreciate the changes made in the manuscript. it is a very interesting manuscript providing background and ideas for future work in the community working with trees.
